# S97. SOCIAL ANXIETY IN SCHIZOPHRENIA: THE IMPACT OF HALLUCINATIONS AND SELF-ESTEEM SUPPORT

**DOI:** 10.1093/schbul/sby018.884

**Published:** 2018-04-01

**Authors:** Laura Faith, Elise Racette, Amber Grove, Melisa Rempfer

**Affiliations:** University of Missouri–Kansas City

## Abstract

**Background:**

Social anxiety is an underreported concern in schizophrenia (SCZ). Prevalence rates in the general population range from 0.5–7% (APA, 2013), but are higher in SCZ, and estimated to be 11–36% (Mazeh et al., 2009; Pallanti et al., 2004). Yet, research is limited with no established social anxiety treatments. Social anxiety is associated with decreased quality of life (Hansson, 2006), low self-esteem (Gumley et al., 2005), and increased psychopathology (Vrbova et al., 2017). Lysaker and Hammersley (2006) found that people with delusions and impairment in flexibility had the highest levels of social anxiety compared to those with fewer symptoms. Additionally, Lysaker et al. (2010) found that people with both high paranoia and theory of mind had higher social anxiety compared to those with lower levels of either paranoia or theory of mind. Taken together, this research suggests that symptoms may increase social anxiety, but other factors may inhibit their impact. The current study aims to add to this literature by exploring how different levels of hallucinations and self-esteem support affect social anxiety in SCZ.

**Methods:**

Outpatients with SCZ (N=50) participated in the current study. Participants were 76% male with a mean age of 42.50. Participants were African-American (n=27; 54%), Caucasian (n=11; 22%), multi-racial (n=5; 10%), Asian (n=4, 8%), or Hispanic (n=3; 6%). Social fear, social avoidance, and overall social anxiety was measured with the Liebowitz Social Anxiety Scale (LSAS; Liebowitz, 1987). Self-esteem support (SeS) was measured with a subscale taken from the Interpersonal Support Evaluation List (ISEL; Cohen & Hoberman, 1983). SeS is the appraisal of the self compared with others and other’s opinions of the self. Hallucinations (HA) were scored with the observer-rated Scale for Assessment of Positive Symptoms (SAPS; Andreasen, 1983). Participants were classified as having hallucinations if their SAPS global hallucinations were rated moderate to severe. This was chosen a priori as it reflects a level of clear hallucinations that may bother the person to some extent, as defined within the SAPS. Participants were classified as having either high or low SeS based on a mean split of the distribution of scores. Once participants were classified, we planned to compare groups on levels of social anxiety. This method was modified from previous research reporting similar groupings of symptoms and their relationship to social anxiety (Lysaker & Hammersley, 2006).

**Results:**

Four groups resulted after including the dichotomized variables with the following proportions: low SeS/no HA (n=6; 12.5%), low SeS/HA (n=11, 22.9%), high SeS/no HA (n=13; 27.1%), and high SeS/HA (n=18, 37.5%). A one-way ANOVA was conducted to analyze the differences between groups. Post-Hoc analyses revealed the following differences. The HA/low SeS group had higher social anxiety than in the no HA/high SeS group (p=.030) and no HA/low SeS group (p=.039). The HA/low SeS group had higher social fear (p=.017) and social avoidance (p=.013) than in the no HA/high SeS group. There was a trending difference revealing that participants in the HA/low SeS group had higher social avoidance than in the HA/high SeS group (p=.056). There was a trending difference revealing that the HA/low SeS group had greater overall social anxiety than those in the HA/high SeS group (p=.064).

**Discussion:**

These results present preliminary findings on social anxiety in people with different levels of HA and SeS. We found that people with low SeS and HA had significantly higher levels of social anxiety, social fear, and social avoidance than participants with only one of neither of these symptoms. These results will be discussed further to highlight implications to treatment and comorbidities in SCZ.

